# Donor-Derived Transmission of *Cryptococcus gattii* sensu lato in Kidney Transplant Recipients

**DOI:** 10.3201/eid2606.191765

**Published:** 2020-06

**Authors:** Daniel W.C.L. Santos, Ferry Hagen, Jacques F. Meis, Marina P. Cristelli, Laila A. Viana, Fabiola D.C. Bernardi, Hélio Tedesco-Silva, José O. Medina-Pestana, Arnaldo L. Colombo

**Affiliations:** Hospital do Rim, São Paulo, Brazil (D. W.C.L. Santos, M.P. Cristelli, L.A. Viana, H. Tedesco-Silva, J.O. Medina-Pestana);; Escola Paulista de Medicina, Universidade Federal de São Paulo, São Paulo (D.W.C.L. Santos, A.L. Colombo);; Canisius Wilhelmina Hospital, Nijmegen, the Netherlands (F. Hagen, J.F. Meis);; Westerdijk Fungal Biodiversity Institute, Utrecht, The Netherlands (F. Hagen);; Centre of Expertise in Mycology Radboudumc/CWZ, Nijmegen, the Netherlands (J.F. Meis);; Faculdade de Ciências Médicas da Santa Casa de São Paulo, São Paulo (F.D.C. Bernardi)

**Keywords:** Antifungal therapy, *Cryptococcus gattii*, donor-transmitted disease, fungal infection, fungi, HIV/AIDS and other retroviruses, immunosuppression, kidney transplantation, Brazil

## Abstract

We describe cases of donor-derived transmission of *Cryptococcus deuterogattii* in 2 kidney transplant recipients in Brazil and published information on other cases. Prompt reduction of immunosuppression and initiation of antifungal therapy was required to successfully control the fungal infections and preserve engraftment.

After antiretroviral therapy for HIV patients was introduced, solid organ transplant recipients became one of the major risk groups for developing cryptococcosis, possibly transmitted from donors (*1*,*2*). We describe 2 cases of donor-derived transmission of *Cryptococcus deuterogattii* in Brazil. The donor in both of these cases was a 43-year-old man with an antemortem history of an unspecified brain tumor who had been declared brain dead after respiratory arrest. 

Case-patient 1 was a 51-year-old man who received a kidney from the donor. Physicians initiated induction therapy with antithymocyte globulin and maintenance therapy with tacrolimus, prednisone, and azathioprine. On day 7 after the procedure, doctors performed a kidney biopsy after the patient experienced delayed graft function. Histopathology of the graft showed organisms consistent with *Cryptococcus* yeast cells, suggesting fungal pyelonephritis. The patient had no respiratory or neurologic complaints. Results from his laboratory tests showed 7,000 leukocytes/mm^3^, 201,000 platelets/μL, serum creatinine 13.29 mg/dL, and serum urea 132 mg/dL. Brain and thorax radiographs revealed no abnormalities. Results of a lumbar puncture showed an opening pressure of 18 cm H_2_O, 2 leukocytes/mm^3^, protein 63 mg/dL, and glucose 77 mg/dL; a *Cryptococcus* antigen latex (CrAg-latex) agglutination test result was positive (titer 1:8). Blood and urine cultures indicated *Cryptococcus* species, and the serum CrAg-latex agglutination test result was positive (titer 1:1,024). 

After the diagnosis of cryptococcosis in the first patient, a 59-year-old woman (case-patient 2) who had received a kidney from the same donor was contacted for evaluation. Physicians had initiated induction therapy with antithymocyte globulin and maintenance therapy with tacrolimus, prednisone, and mycophenolic acid. She was discharged on day 8 after the procedure and was asymptomatic when recalled on day 10. Clinicians performed blood and urine cultures, radiographs of the chest and the brain, and a lumbar puncture. The radiograph revealed no abnormalities. Results of lumbar puncture showed opening pressure of 15 cm H_2_O, 3 leukocytes/mm^3^, protein 35 mg/dL, and glucose 174 mg/dL. Results of India ink and CrAg-latex agglutination tests were positive (titer: 1:64). Blood cultures and a urine sample indicated *Cryptococcus* species. No fungal growth was detected in a cerebrospinal fluid sample. The serum CrAg-latex agglutination was positive (titer 1:1,024). 

A revised histology of the brain biopsy of the donor showed yeasts of *Cryptococcus* species that had not been detected previously. Results of a CrAg-latex agglutination test performed on a stored serum sample from the donor was positive (titer: 1:1,024). To rule out infection by *Cryptococcus* species in the organ recipients prior to the transplantations, CrAg-latex agglutination tests were performed on stored pretransplant serum samples from both recipients; results for both were negative. 

Both patients were treated with amphotericin B lipid complex (5 mg/kg 1×/d for 21 d) in combination with 5-fluorocytosine (25 mg/kg 4×/d for 14 d); dosages of immunosuppressive drugs were lowered. Therapy was switched to intravenous fluconazole (400 mg/d) after results of blood and urine cultures became negative. After clinical and microbiological remission of the infection, dosage was adjusted on the basis of renal function to 300 mg/d of oral fluconazole and was maintained for 2 years. Three years after stopping all antifungal therapy, no relapse of cryptococcosis was documented. 

All 6 isolates from the recipients were characterized at a reference laboratory at Universidade Federal de São Paulo (São Paulo, Brazil) and sent to Canisius-Wilhelmina Hospital (Nijmegen, The Netherlands). We performed AFLP (amplified fragment length polymorphism) fingerprinting and multilocus sequencing typing, which showed that all isolates were *C. deuterogattii* genotype AFLP6/VGII. The revision of slides from formalin-fixed paraffin-embedded tissue blocks from donor brain biopsies showed *Cryptococcus* yeasts. We performed fungal DNA extraction and amplified parts of *CAP59*, *GPD1*, *IGS1*, *LAC1*, *PLB1*, *SOD1*, and *URA5* loci (*2*). Phylogenetic analysis showed genetic similarity between the *C. deuterogattii* isolates from the kidney recipients and those from the donor’s brain (Figure). 

**Figure:**
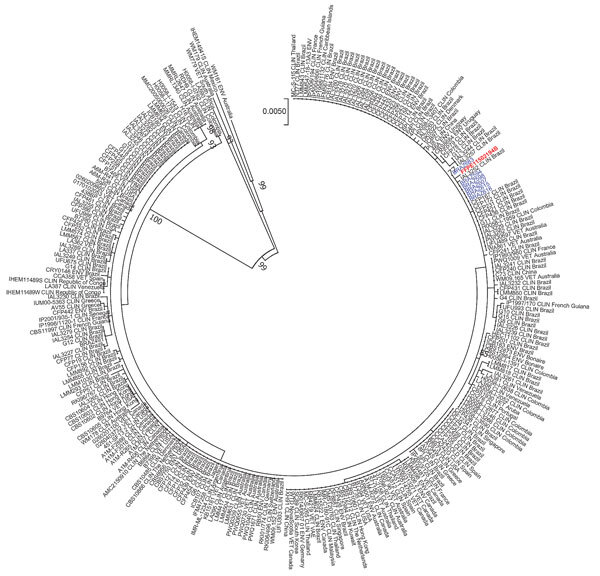
Phylogenetic maximum-likelihood analysis of 6 *Cryptococcus deuterogattii* isolates from 2 kidney transplant recipients in Brazil (blue), the organ donor (red), and cases from the literature (Appendix, https://wwwnc.cdc.gov/EID/article/26/6/19-1765-App1.pdf), along with reference isolates. Isolates from the 2 transplant recipients came from blood and urine; the genetic material from the donor came from a formalin-fixed paraffin-embedded brain tissue block. The GenBank accession numbers are KU642696-KU642701 (*CAP59*), KU642741-KU642746 (*GPD1*), KU642786-KU642791 (IGS1), KU642831-KU642836 (*LAC1*), KU642876-KU642881 (*PLB1*), KU642967-KU642972 (*SOD1*) and KU642921-KU642926 (*URA5*). Tree represents 1,000x bootstraps. Scale bar represents substitutions per site.

We searched the literature for other cases of fungal infections from solid organ donors in transplant recipients and found 12 additional cases of presumed or confirmed donor-derived cryptococcosis (Appendix). Molecular identification was provided in only 6 out of 14 graft recipients (*3*–*10*). On the basis of the 2 cases we describe and data from the literature review, we suggest the following procedures for effective clinical management: performing blood and urine cultures, radiograph of the lungs and brain, and lumbar puncture to rule out dissemination; reducing immunosuppression to control infection; promptly initiating induction combination therapy with an amphotericin B lipid complex and 5-fluorocytosine; preserving the infected engraftment, if possible, in the absence of large fungal masses and abscesses; and extending the length of antifungal therapy when fungal elements persist in tissues. 

In conclusion, we found that *C. deuterogattii* may be transmitted by infected allografts, representing a medical concern in countries to which *C. gattii* species complex is endemic. Cryptococcosis incidence might be reduced by excluding organ donor candidates with a history of neurologic disease without a clear definition of its etiology. Ensuring that clinicians are trained to recognize and treat this fungal infection would likely further reduce transmission from donors. 

Appendix Demographic, clinical, and transplant data of donor-derived transmission of confirmed and presumed *Cryptococcus* spp. in solid organ transplantation.

## References

[1] Baddley JW, Forrest GN; AST Infectious Diseases Community of Practice. Cryptococcosis in solid organ transplantation-Guidelines from the American Society of Transplantation Infectious Diseases Community of Practice. Clin Transplant. 2019;33:e13543. 10.1111/ctr.13543 30900315

[2] Hagen F, Khayhan K, Theelen B, Kolecka A, Polacheck I, Sionov E, et al. Recognition of seven species in the *Cryptococcus gattii*/*Cryptococcus neoformans* species complex. Fungal Genet Biol. 2015;78:16–48. 10.1016/j.fgb.2015.02.009 25721988

[3] Ooi BS, Chen BT, Lim CH, Khoo OT, Chan DT. Survival of a patient transplanted with a kidney infected with *Cryptococcus neoformans.* Transplantation. 1971;11:428–9. 10.1097/00007890-197104000-00018 4934356

[4] Beyt BE Jr, Waltman SR. Cryptococcal endophthalmitis after corneal transplantation. N Engl J Med. 1978;298:825–6. 10.1056/NEJM197804132981506 345120

[5] Kanj SS, Welty-Wolf K, Madden J, Tapson V, Baz MA, Davis RD, et al. Fungal infections in lung and heart-lung transplant recipients. Report of 9 cases and review of the literature. Medicine (Baltimore). 1996;75:142–56. 10.1097/00005792-199605000-00004 8965683

[6] de Castro LE, Sarraf OA, Lally JM, Sandoval HP, Solomon KD, Vroman DT. *Cryptococcus albidus* keratitis after corneal transplantation. Cornea. 2005;24:882–3. 10.1097/01.ico.0000157404.34774.1a 16160511

[7] Baddley JW, Schain DC, Gupte AA, Lodhi SA, Kayler LK, Frade JP, et al. Transmission of *Cryptococcus neoformans* by organ transplantation. Clin Infect Dis. 2011;52:e94–8. 10.1093/cid/ciq216 21220771

[8] MacEwen CR, Ryan A, Winearls CG. Donor transmission of *Cryptococcus neoformans* presenting late after renal transplantation. Clin Kidney J. 2013;6:224–7. 10.1093/ckj/sft006 26019853 PMC4432446

[9] Chang CM, Tsai CC, Tseng CE, Tseng CW, Tseng KC, Lin CW, et al. Donor-derived *Cryptococcus* infection in liver transplant: case report and literature review. Exp Clin Transplant. 2014;12:74–7. 10.6002/ect.2012.0288 23901902

[10] Camargo JF, Simkins J, Schain DC, Gonzalez AA, Alcaide ML, Anjan S, et al. A cluster of donor-derived *Cryptococcus neoformans* infection affecting lung, liver, and kidney transplant recipients: Case report and review of literature. Transpl Infect Dis. 2018;20:e12836. 10.1111/tid.12836 29359837

