# Supplementary material for: Donor-Derived Transmission of Cryptococcus gattii sensu lato in Kidney Transplant Recipients
Source: Emerg Infect Dis. 2020 Jun;26(6):1329–31. doi: 10.3201/eid2606.191765 (PMC7258454; doi:10.3201/eid2606.191765)
Supplement: Appendix — Demographic, clinical, and transplant data of donor-derived transmission of confirmed and presumed Cryptococcus spp. in solid organ transplantation. [file 19-1765-Techapp-s1.pdf]

Article DOI: https://doi.org/10.3201/eid2606.191765

Donor-Derived Transmission of *Cryptococcus gattii* sensu lato in Kidney Transplant Recipients

Appendix

Appendix Table. Demographic, clinical, and transplant data of donor-derived transmission of confirmed and presumed *Cryptococcus* spp. in solid organ transplantation

Reference no.<br>Sex/age of recipient<br>Type of transplant<br>Transmission category	Characteristics of the donor	Time to infection diagnosis after transplant	Clinical and laboratorial findings of recipient's fungal diagnosis	Treatment	Outcome
NA (case 1 this study)<br>M/51 y<br>Kidney, deceased donor<br>Confirmed transmission	M/43 y with unspecified brain tumor diagnosed before death. Autopsy showed brain cryptococcoma. FFPE material subjected to molecular testing, which identified *C. deuterogattii*.	7 d	Delayed graft function and hemodialysis. Blood and urine cultures yielded *C. deuterogattii*. CSF positive for cryptococcal antigen (1:8)	Lipid complex AMB plus 5-FC, switched to oral FLU	Alive
NA (case 2 this study)<br>F/59 y<br>Kidney, deceased donor<br>Confirmed transmission	M/43 y with unspecified brain tumor diagnosed before death. Autopsy showed brain cryptococcoma. FFPE material subjected to molecular testing, which identified *C. deuterogattii*.	10 d	Asymptomatic. Blood and urine cultures yielded *C. deuterogattii*. CSF positive for cryptococcal antigen (1:64)	Lipid complex AMB plus 5-FC, switched to oral FLU	Alive
(3)<br>F/29 y<br>Kidney, deceased donor<br>Confirmed transmission	M/43 y with presumed diagnosis of brain tumor and intracranial hypertension. Donor kidney not transplanted showed 2 cryptococcal granulomas.	5 d	Asymptomatic. Urine culture yielded *Cryptococcus* spp. Chest radiograph was normal, CSF testing was not performed.	Conventional AMB plus 5-FC	Alive at 10 wk after transplant, negative urine cultures
(4)<br>F/8 y<br>Cornea, deceased donor<br>Confirmed transmission	F/25 y with polymyositis. Persistent fever, died due to progressive respiratory failure. Blood cultures yielded *C. neoformans.* Postmortem exam revealed generalized cryptococcosis.	2 mo	Decreased visual acuity with a 3 mm yellow-white mass in anterior chamber. Aqueous fluid with positive culture for *C. neoformans* and a positive antigen (1:512)	Topical and systemic conventional AMB and 5-FC for 6 wk	Alive at 2 months after transplant, better light perception, only residual corneal clouding in affected eye
(5)<br>F/24 y<br>Lung, deceased donor<br>Presumed transmission	Not reported	2 d	2 d after operation, recipient had fever, leukocytosis, and hypoxemia. Endotracheal cultures yielded *C. neoformans*. Serum cryptococcal antigen and blood cultures were negative. CSF testing was not performed.	FLU (400 mg/day) for 4 mo	Alive at 12 mo after transplant
(6)<br>F/69 y<br>Cornea, deceased donor<br>Presumed transmission	F/58 y	7 mo	Persistent corneal button edema that required sutures 5 d after operation. The edema persisted and 9 mo later second corneal transplant was performed by removing the corneal	Curative surgery	Alive and cured after the 2nd corneal transplant

Reference no.<br>Sex/age of recipient<br>Type of transplant<br>Transmission category	Characteristics of the donor	Time to infection diagnosis after transplant	Clinical and laboratorial findings of recipient's fungal diagnosis	Treatment	Outcome
			button. Culture of corneal tissue yielded *Naganishia albidus* (formerly *C. albidus*).		
(7)<br>Recipient 1: F/72 y<br>Liver, deceased donor<br>Confirmed transmission	F/51 y with sarcoidosis. Headache, slurred speech and hydrocephalus. Autopsy available after 30 d after transplant showed meningoencephalitis by *C. neoformans*.	2 wk	Gastrointestinal bleeding and immune thrombocytopenic purpura. Histopathology of liver and spleen showed *Cryptococcus* spp. Blood cultures, liver and spleen tissues yielded *C. neoformans*. CSF testing was not performed.	Lipid formulation of AMB for 8 wk, switched to FLU	Death due to aspiration pneumonia 6 mo after transplant
(7)<br>Recipient 2: M/58 y<br>Kidney, deceased donor<br>Confirmed transmission	F/51 y with sarcoidosis. Headache, slurred speech and hydrocephalus. Autopsy available after 30 d after transplant showed meningoencephalitis by *C. neoformans*.	16 d	Fever, malaise. Blood cultures yielded *C. neoformans* and serum cryptococcal antigen (1:256). CSF analysis was normal with cryptococcal antigen negative. Chest radiograph was performed showing focal consolidation and effusions.	Lipid formulation of AMB and 5-FC for 14 d switched to FLU	Alive for 6 mo with oral FLU
(7)<br>Recipient 3: M/46 y<br>Kidney, deceased donor<br>Confirmed transmission	F/51 y with sarcoidosis. Headache, slurred speech and hydrocephalus. Autopsy available after 30 d after transplant showed meningoencephalitis by *C. neoformans*.	24 d	Fever, neck stiffness, and photophobia. CSF and blood cultures yielded *C. neoformans*.	Lipid formulation of AMB and 5-FC for 17 d switched to FLU	Alive for 6 mo with oral FLU
(8)<br>M/50 y<br>Kidney, deceased donor<br>Confirmed transmission	Donor died of presumed bacterial meningitis.<br>*C. neoformans* yielded from donor CSF and blood after the transplant.	9 wk	Nausea, vomiting, severe headache, coughing, and respiratory effort. CSF cultures yielded *C. neoformans* and CSF cryptococcal antigen was positive.	Liposomal AMB and intravenous 5-FC for 3 wk, switched to oral FLU	Alive for 9 mo with oral FLU
(9)<br>F/63 y<br>Liver, deceased Donor<br>Presumed transmission	M/48 y with severe intracranial hemorrhage. CSF testing was not performed.	≤1 wk	Dyspnea and respiratory failure, fever, and rising bilirubin.<br>Blood cultures yielded *C. neoformans* and serum antigen serum was positive (1:2048). Serum cryptococcal antigen test before transplant was negative.	Conventional AMB followed by liposomal AMB for 4 wk, switched to oral FLU	Alive for 1.5 y after transplant
(10)<br>Recipient 1<br>F/42 y<br>Lung, deceased donor<br>Confirmed transmission	M/55 y with nausea, vomiting, mental status deteriorated and brain death. Radiograph of the chest revealed right lobe infiltrate. 5 d after transplant blood and BAL cultures yielded *C. neoformans*.	Immediately after operation	Fever. Recipient's BAL culture yielded *C. neoformans*.<br>Blood culture and serum cryptococcal antigen were negative.	VOR ≤90 d after transplant, switched to lipid complex AMB for 12 d, on discharge switched to POS	Alive for ≥10 mo, negative BAL cultures
(10)<br>Recipient 2<br>M/77 y<br>Kidney, deceased donor<br>Confirmed transmission	M/55 y with nausea, vomiting, mental status deteriorated and brain death. Radiograph of the chest revealed right lobe infiltrate. 5 d after transplant blood and BAL cultures yielded *C. neoformans*.	67 d	Fever, weakness, gait disturbance, and confusion. CSF positive for cryptococcal antigen (1:1280) and CSF positive culture for *Cryptococcus* spp.	Lipid complex AMB plus 5-FC, switched to oral FLU	Alive

Reference no.<br>Sex/age of recipient<br>Type of transplant<br>Transmission category	Characteristics of the donor	Time to infection diagnosis after transplant	Clinical and laboratorial findings of recipient's fungal diagnosis	Treatment	Outcome
(10)<br>Recipient 3<br>M/58 y<br>Liver, deceased donor<br>Confirmed transmission	M/55 y with nausea, vomiting, mental status deteriorated and brain death. Radiograph of the chest revealed right lobe infiltrate. 5 d after transplant blood and BAL cultures yielded *C. neoformans*.	106 d	Dizziness, headache, diplopia, blurred vision, and gait disturbance. Blood and CSF cultures yielded *C. neoformans.*	Lipid complex AMB plus 5-FC	Death due to complications from intracranial hypertension

*References provided in the main text of the article. CSF, cerebrospinal fluid; FFPE, formalin-fixed paraffin-embedded; NA, not applicable; 5-FC, 5-fluorocytosine; AMB, amphotericin; FLU, fluconazole; POS, posaconazole; VOR, voriconazole.
